# Editorial: Advances and Current Challenges in Calcium Signaling Within the Cardiovascular System

**DOI:** 10.3389/fphys.2021.696315

**Published:** 2021-07-23

**Authors:** Roberto Berra-Romani, Germano Guerra, Francesco Moccia

**Affiliations:** ^1^Department of Biomedicine, School of Medicine, Benemérita Universidad Autónoma de Puebla, Puebla, Mexico; ^2^Department of Medicine and Health Sciences “V. Tiberio”, University of Molise, Campobasso, Italy; ^3^Laboratory of General Physiology, Department of Biology and Biotechnology “L. Spallanzani”, University of Pavia, Pavia, Italy

**Keywords:** excitation-contracting coupling, endothelial signaling, heart failure, cardiac arrhtyhmias, phenotypic swich, angiogenesis, vascular permeability

Calcium signaling in the cardiovascular system does not only drive “excitation-contraction” coupling in cardiomyocytes and vascular smooth muscle cells (VSMCs) (Eisner et al., 2017). Intracellular Ca^2+^ signals finely tune endothelial functions, thereby regulating also vascular tone and permeability, angiogenesis and vasculogenesis, coagulation and inflammation (Smani et al., 2018). An increase in intracellular Ca^2+^ concentration ([Ca^2+^]_i_) can be achieved through the concerted interaction among the components of a versatile network of membrane receptors, ion channels, transporters and buffers that can be uniquely assembled by each cell type to produce distinct spatio-temporal Ca^2+^ signals that are selectively tailored to adjust specific cardiovascular functions (Berridge, 2003). It is, therefore, not surprising that rewiring of the Ca^2+^ handling machinery drives or is intimately involved in many cardiovascular diseases, including cardiac hypertrophy, chronic heart failure, ventricular and atrial arrhythmias, vascular proliferative disorders (atherosclerosis and hypertension), and diabetes (Berridge, 2003; Moccia et al., 2019).

Three of the six contributions of this Research Topic are original research articles that shed novel light on the pathogenic role of deranged Ca^2+^ signaling in VSMCs and cardiac myocytes and describe novel strategies to rescue cardiovascular function by dampening such aberrant elevations in [Ca^2+^]_i_. Lopez et al. reported about the first description of dysregulated intracellular Ca^2+^ homeostasis in VSMCs deriving from an established murine model of Duchenne syndrome, known as mdx mice. These authors found that [Ca^2+^]_i_ and intracellular Na^+^ concentration ([Na^+^]_i_) were both increased in mdx VSMCs. Furthermore, they found that cyclic stretch induced a larger elevation in [Ca^2+^]_i_ and in [Na^+^]_i_ in mdx VSMCs as compared to wild type (WT) controls. Pharmacological analysis led the Authors to hypothesise that mechanical stimulation is sensed by G-protein coupled receptors, thereby leading to the intracellular production of diacylglycerol, followed by the activation of Transient Receptor Potential Canonical 1 (TRPC1), TRPC3, and TRPC6. Intriguingly, all these channels were upregulated and associated to higher membrane fragility in mdx VSMCs as compared to WT cells. Thus, TRPC1, TRPC3, and TRPC6 could be regarded as novel targets to prevent VSMC dysfunction in Duchenne syndrome.

Wong et al. examined the effect of topographical (microgroove-induced alignment, μ), hormonal [triiodothyronine (T3) induction], and electrical (electrical conditioning, EC) cues on the biophysical and Ca^2+^-handling properties of human embryonic stem cell-derived ventricular cardiomyocytes (hESC-VCMs). High-resolution optical mapping revealed that combinatorial application of μ, T3 and EC remarkably increased the conduction velocity, anisotropic ratio, and percentage of mature quiescent-yet-excitable preparations. This pro-maturational effect of combined μ-T3-EC on hESC-VCMs was associated to the upregulation of *SCN1B* transcripts, and the consequent increase in the current density of the voltage-gated Na^+^ current, while the *HCN2/4* transcripts and associated funny current were downregulated. Finally, the combined μ-T3-EC strategy boosted the maturation of the Ca^2+^ cycling machinery hESC-VCMs due to the upregulation of the transcripts encoding for transcripts encoding for sarco/endoplasmic reticulum Ca^2+^-ATPase (SERCA) and phospholamban transcripts. This strategy, therefore, paves the way for future investigations exploiting hESC-VCMs for cardiac disease modelling and *in vitro* drug screening.

Di Mauro et al. proposed a novel pharmacological approach to treat Brugada syndrome (BrS), an inherited arrhythmogenic disease that can cause sudden death in young individuals. Approximately 12% of BrS cases are due loss-of-function mutations in the *CACNA1C* and *CACNB2* genes, which, respectively, encode for the α- (Ca_v_α1.2) and β2- (Ca_v_β2) subunits of L-type Ca^2+^ channels in cardiac myocytes (Napolitano and Antzelevitch, 2011). Di Mauro et al. detected two novel BrS-related Ca_v_α1.2 mutations (T320M and Q428E) which impaired Ca_v_β2-mediated trafficking of Ca_v_α1.2 to the plasma membrane and thereby reduced voltage-gated Ca^2+^ currents in a heterologous expression system. By using a mimetic peptide targeting the COOH-terminal tail of Ca_v_β2, these Authors were able to restore Ca_v_α1.2 trafficking to the plasma membrane, which resulted in voltage-gated Ca^2+^ currents similar to the WT isoform. This mimetic peptide could, therefore, represent a promising tool to treat BrS patients, although it remains to be probed in cardiac myocytes bearing these BrS-related Ca_v_α1.2 mutations.

The remaining three articles of these Research Topic are reviews and perspective articles mainly dealing with the endothelial Ca^2+^ toolkit.

Genova et al. discussed the role played by several TRP channel isoforms in the regulation of endothelial permeability along the vascular tree. These Authors first discussed how some TRP channels, i.e., TRPC1 and TRPC4, may serve in a store-dependent manner upon their physical association with STIM1 and/or Orai1 to regulate the endothelial barrier function (Moccia et al., 2012). Then, they described the contribution of other endothelial TRP channels, e.g., TRPC3, TRPC4, TRP vanilloid 1 (TRPV1), TRPV4, and TRP melastatin 4 (TRPM4), in the regulation of vascular permeability. The Authors conclude that the lack of a detailed knowledge of TRP channel expression and downstream effectors in arterial/vein as compared to capillary endothelial cells is a major limitation to understand how TRP channels contribute to modulate vascular permeability.

Negri et al. described the role played by endothelial TRP channels in vascular remodelling by focusing on three main processes: angiogenesis and arteriogenesis, which are effected by vascular endothelial cells, and vasculogenesis, which is effected by endothelial colony forming cells. These Authors focused, therefore, their attention on TRPC1, TRPC3, TRPC4, TRPC5, TRPC6, TRPV1, TRPV4, TRPM2, TRPM4, TRPM7, TRP ankyrin 1 (TRPA1). Furthermore, the role of endothelial TRP channels in tumour vascularization by endothelial cells and ECFCs was also described. These processes have been mainly associated to TRPC1, TRPC3, TRPV2, TRPV4, TRPM8, and TRPA1.

The last review article of this Research Topic was by Moccia et al. who discussed the possibility to target endolysosomal two-pore channels (TPCs) to treat cardiovascular disorders in patients affected by the novel COronaVirus Disease 2019 (COVID-19). Early work showed that TPCs could be targeted to prevent Ebola virus and Middle East Respiratory Syndrome COronaVirus (MERS-CoV) infectivity (Chao et al., 2020). Therefore, the Authors speculate that TPCs also mediate severe acute respiratory syndrome coronavirus type 2 entry into cardiovascular cells, which widely express the cognate ACE2 receptor. Of note, many Food and Drug Administration-approved compounds inhibit TPCs and prevent MERS-CoV/Ebola virus infection of host cells, which may potentially expand our capability to treat COVID-19 patients with already available drugs.

## Author Contributions

FM drafted the manuscript and supervised the work. All authors contributed to the preparation of the manuscript and approved the submitted version.

## Conflict of Interest

The authors declare that the research was conducted in the absence of any commercial or financial relationships that could be construed as a potential conflict of interest.

## Publisher's Note

All claims expressed in this article are solely those of the authors and do not necessarily represent those of their affiliated organizations, or those of the publisher, the editors and the reviewers. Any product that may be evaluated in this article, or claim that may be made by its manufacturer, is not guaranteed or endorsed by the publisher.
